# Supplementary material for: High-Throughput 1H-Nuclear Magnetic Resonance-Based Screening for the Identification and Quantification of Heartwood Diterpenic Acids in Four Black Pine (Pinus nigra Arn.) Marginal Provenances in Greece
Source: Molecules. 2019 Oct 7;24(19):3603. doi: 10.3390/molecules24193603 (PMC6804012; doi:10.3390/molecules24193603)
Supplement: Supplementary file 1 [file molecules-24-03603-s001.pdf]

molecules

MDPI

Article

High-Throughput ^{1}H-Nuclear Magnetic Resonance-based screening for the identification and quantification of heartwood diterpenic acids in four Black pine (*Pinus nigra* Arn.) marginal provenances in Greece.

Kostas Ioannidis [1,2]*, Eleni Melliou [2] and Prokopios Magiatis [2]

[1] Laboratory of Forest Genetics and Biotechnology, Institute of Mediterranean and Forest Ecosystems, Hellenic Agricultural Organization "Demeter", Ilissia, 11528 Athens, Greece; ioko@fria.gr

[2] Department of Pharmacognosy and Natural Products Chemistry, Faculty of Pharmacy, University of Athens, Panepistimiopolis Zografou, Athens 15771, Greece; emelliou@pharm.uoa.gr (E.M.); magiatis@pharm.uoa.gr (P.M.) ; ioko@fria.gr (K.I.)

* Corresponding author: ioko@fria.gr (K.I.); Tel.: +30-210-77-83-750; Fax: +30-210-77-84-602

Received: date; Accepted: date; Published: date

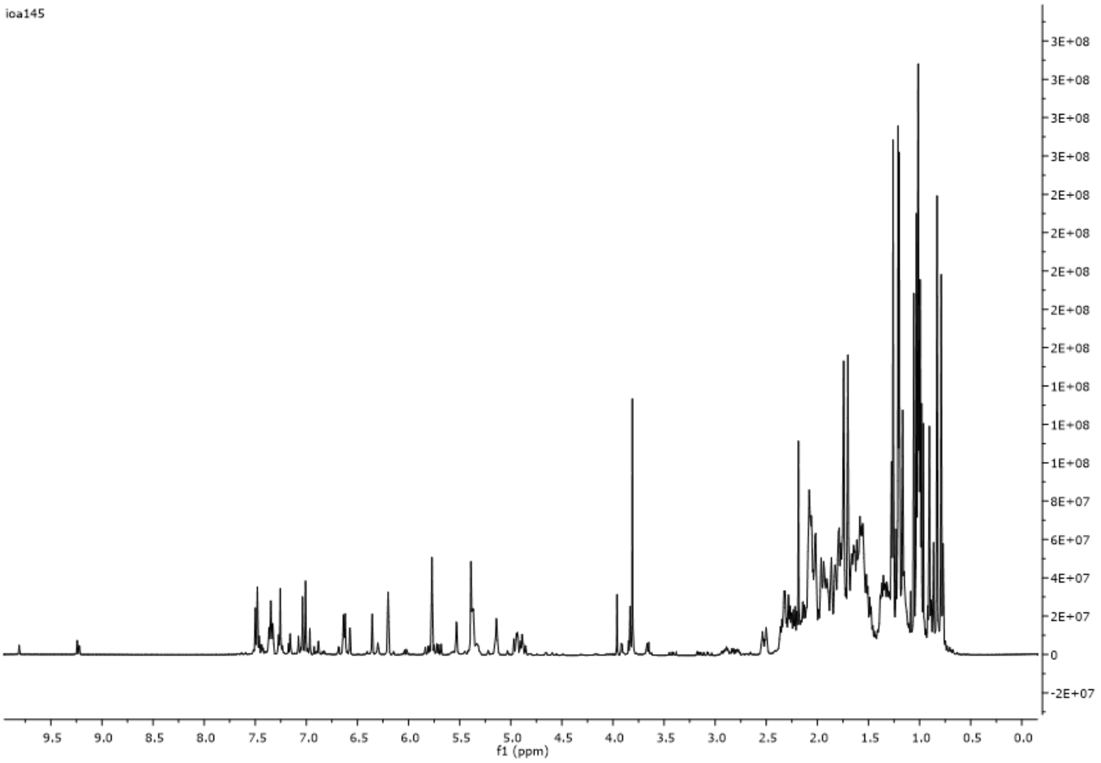

Figure S1. The ^{1}H-NMR spectrum (400MHz, $CDCl_3$, δ-values in ppm) of Black pine (*P. nigra* Arn.) heartwood extract.

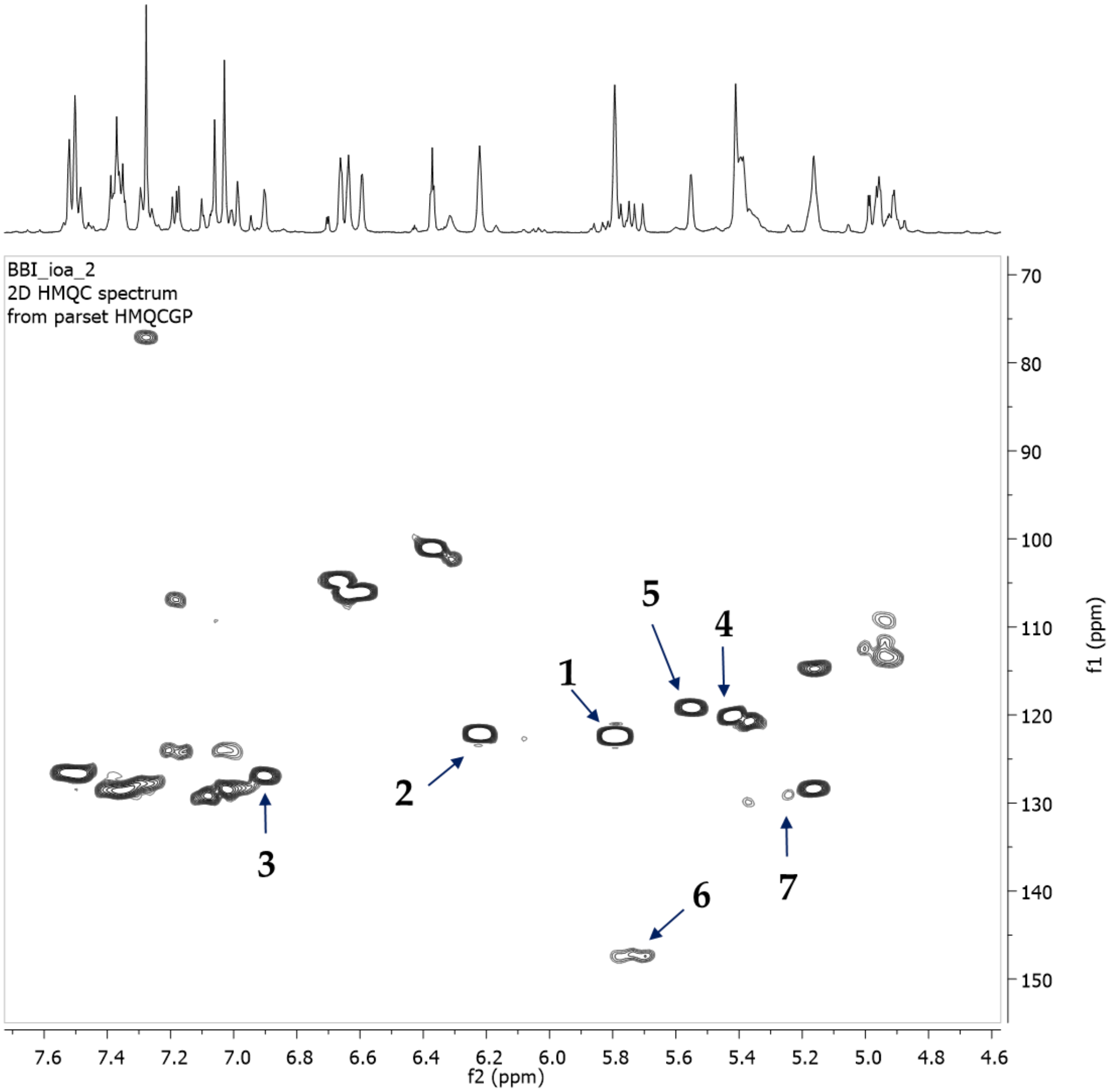

Figure S2. 2D HMQC spectrum (400MHz, $CDCl_3$, δ-values in ppm) of Black pine (*P. nigra* Arn.) heartwood extract with low concentration of isopimaric acid (**8**).

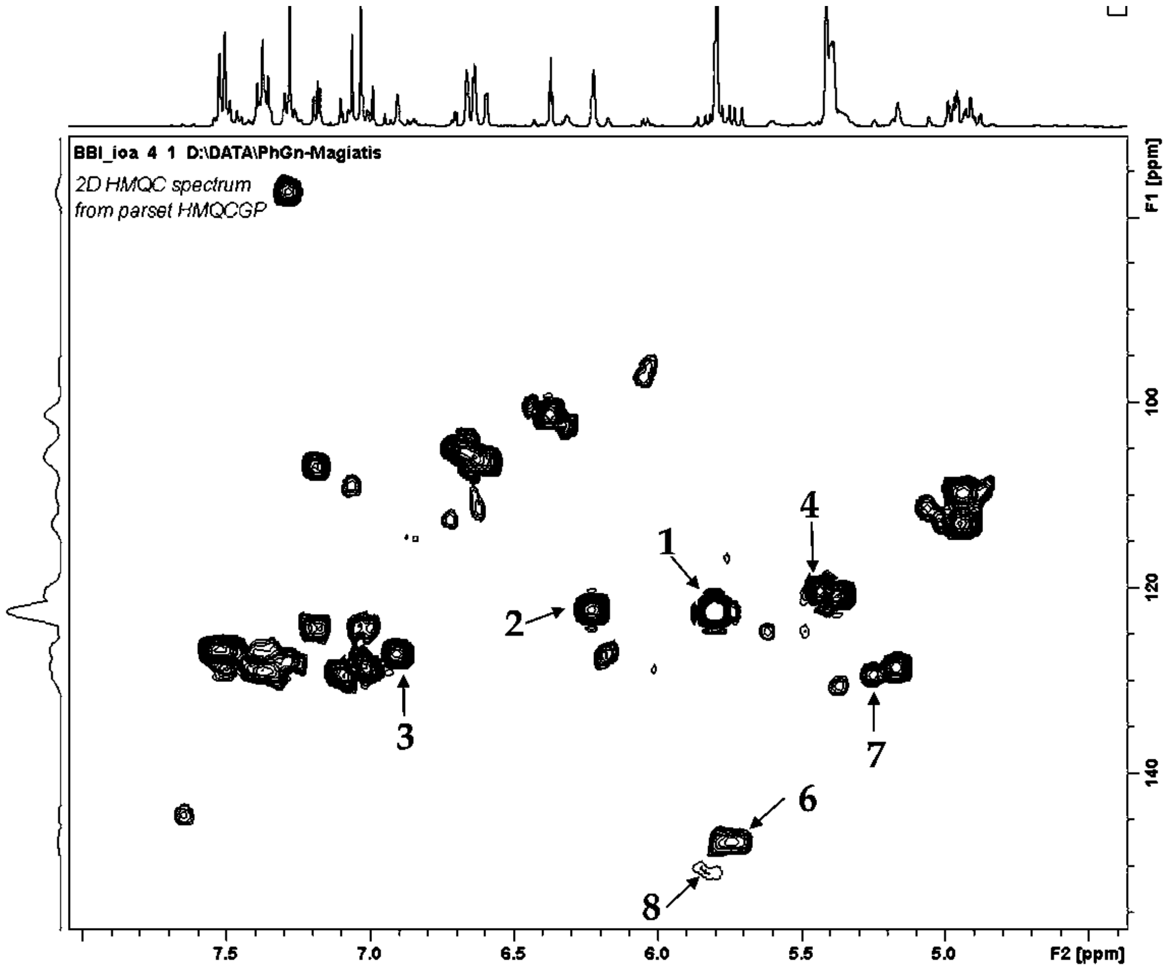

Figure S3. The 2D HMQC ¹H-NMR spectrum (400MHz, $CDCl_3$, δ-values in ppm) of Black pine (*P. nigra* Arn.) heartwood extracts with low concentration of levopimaric acid (**5**).

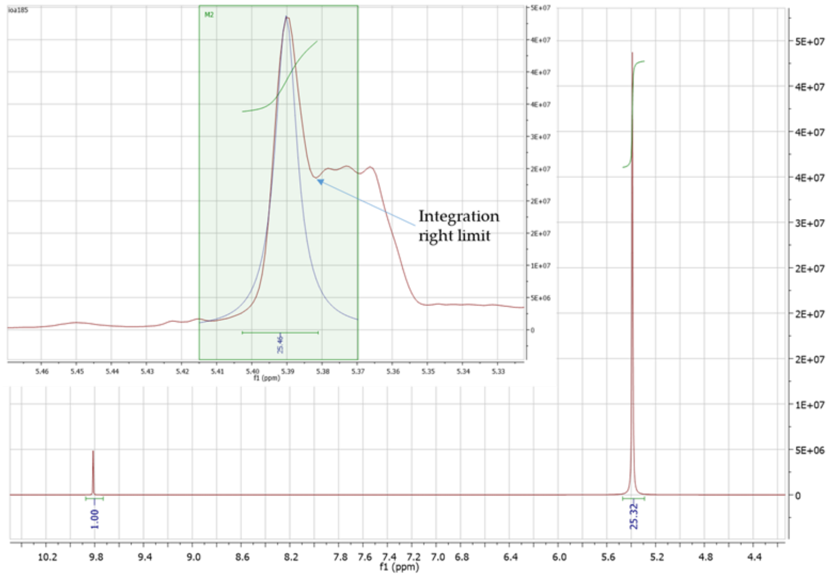

Figure S4. Deconvolution of a spectrum (400MHz, $CDCl_3$, δ-values in ppm) in order to quantify the partial overlapping of peak 4.

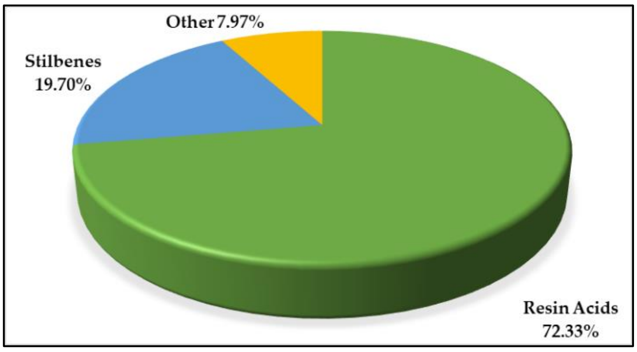

Figure S5. The major components of acetone extractives.

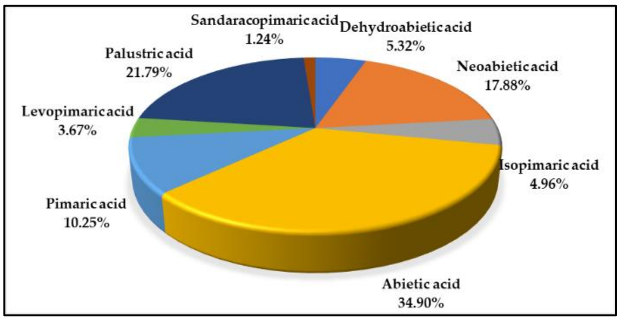

Figure S6. The mean amounts of each individual resin acid.

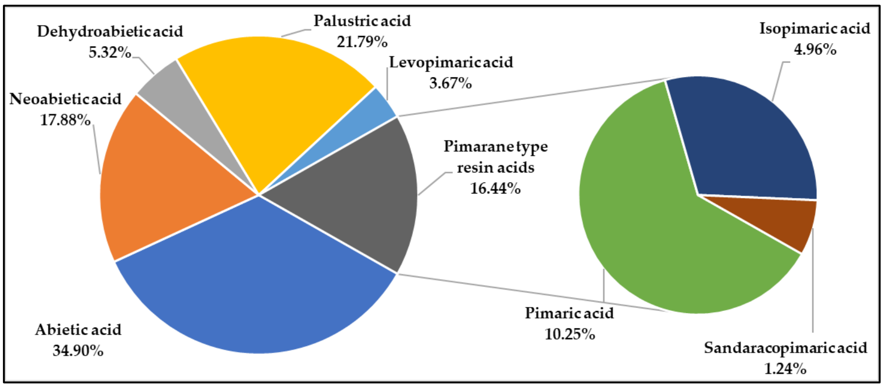

Figure S7. The mean percentage of abietane and pimarane types of resin acids.

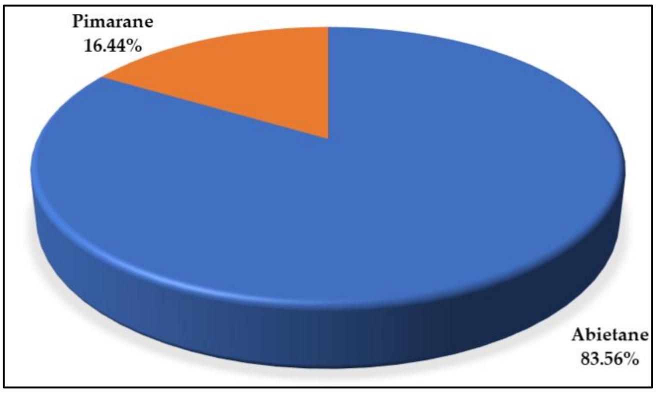

Figure S8. The mean percentage composition of the two resin acids types, i.e. abietane and pimarane.

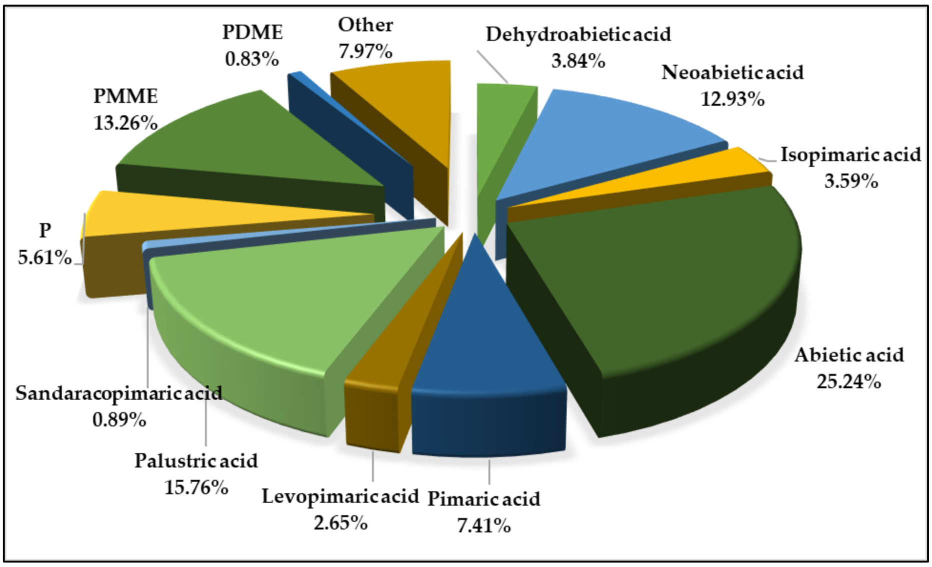

Figure S9. The mean percentage content of the constituents in the Black pine's heartwood acetone extraction from the Peloponnese (P= pinosylvin, PMME and PDME = monomethylether and dimethylether of pinosylvin respectively).

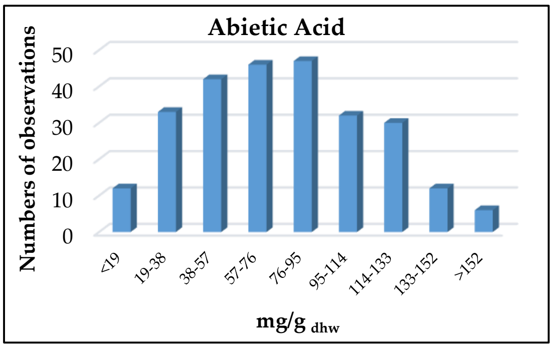

(**a**)

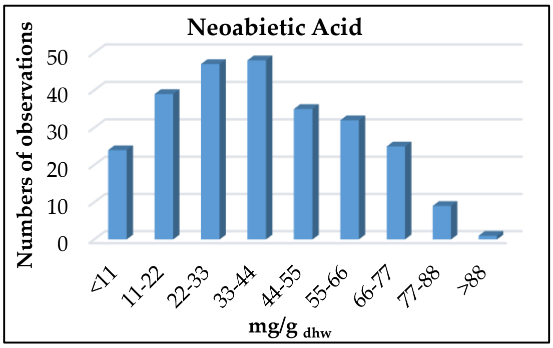

(**b**)

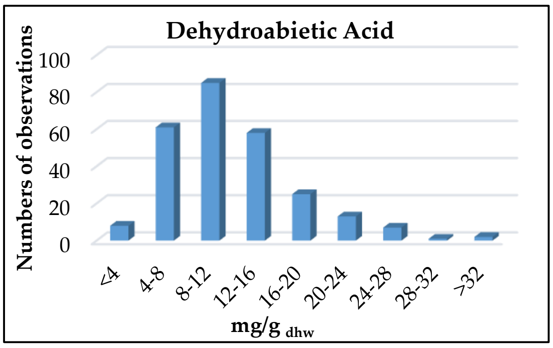

(c)

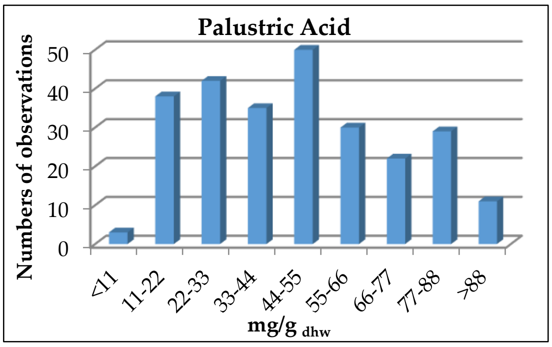

(d)

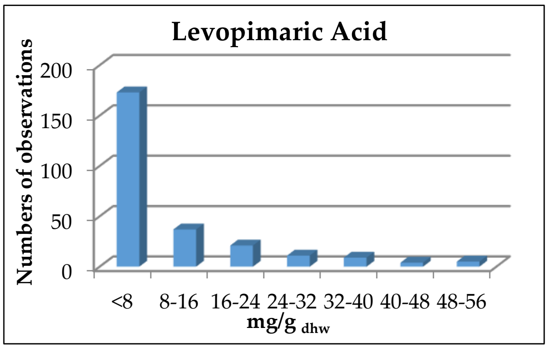

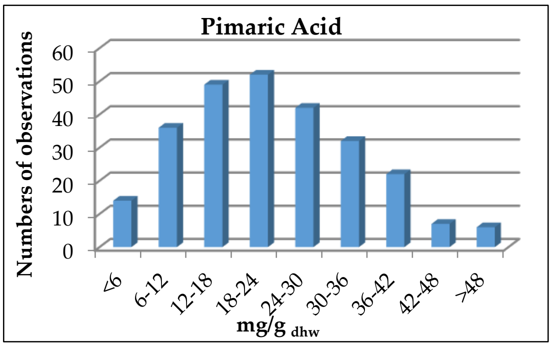

(e)

(f)

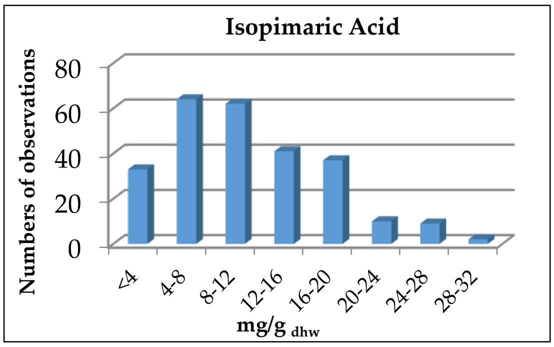

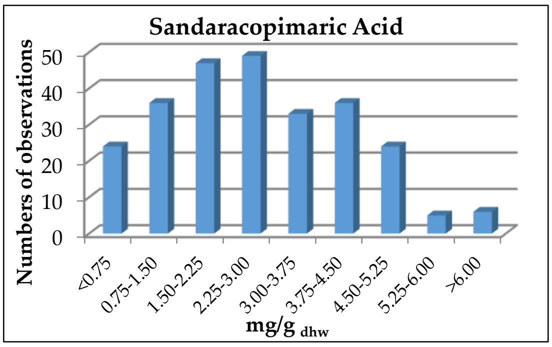

(g)

(h)

Figure S10. Resin acids frequency distribution of Black pine (*P. nigra* Arn.): (a) abietic acid; (b) neoabietic acid; (c) dehydroabietic acid, (d) palustric acid, (e) levopimaric acid, (f) pimaric acid, (g) isopimaric acid and (h) sandaracopimaric acid.

Table S1. Precision data.

**Precision**													
**ID**	**NMR Spectrum**	**Sampling Site**	**Provenance**	**Clone**	**Replication**	**Abietic acid**	**Neoabietic acid**	**Dehydroabietic acid**	**Palustric acid**	**Levopimaric acid**	**Pimaric acid**	**Sandaracopimaric acid**	**Isopimaric acid**
799-21B	Ioa330	3	Feneos	21	1	8.05	0.99	2.63	3.88	0.18	0.86	0.24	0.25
799-21B	Ioa331	3	Feneos	21	2	7.09	0.90	2.17	3.80	0.20	0.76	0.19	0.23
799-21B	Ioa332	3	Feneos	21	3	7.93	1.05	2.64	3.68	0.16	0.80	0.23	0.20
					Average	7.69	0.98	2.48	3.79	0.18	0.81	0.22	0.23
					Standard Deviation (s)	0.4271	0.0616	0.2192	0.0822	0.0163	0.0411	0.0216	0.0205
					**Coefficient of Variance (CV)**	**5.55**	**6.29**	**8.84**	**2.17**	**9.07**	**5.09**	**9.82**	**9.07**
578-36A	Ioa333	2	Parnonas	36	1	12.48	2.26	5.02	6.94	0.42	1.68	0.42	1.47
578-36A	Ioa334	2	Parnonas	36	2	14.28	2.65	5.46	7.87	0.45	2.02	0.45	1.49
578-36A	Ioa335	2	Parnonas	36	3	12.47	2.29	5.32	7.55	0.40	1.83	0.43	1.38
					Average	13.08	2.40	5.27	7.45	0.42	1.84	0.43	1.45
					Standard Deviation (s)	0.8509	0.1772	0.1835	0.3858	0.0205	0.1391	0.0125	0.0478
					**Coefficient of Variance (CV)**	**6.51**	**7.38**	**3.49**	**5.18**	**4.85**	**7.55**	**2.88**	**3.31**
821-47B	Ioa336	3	Taigetos	47	1	14.26	8.70	2.24	8.44	0.54	1.08	0.62	0.54
821-47B	Ioa337	3	Taigetos	47	2	13.73	8.44	2.38	10.61	0.67	1.29	0.66	0.61
821-47B	Ioa338	3	Taigetos	47	3	13.03	7.65	2.32	10.38	0.60	1.26	0.62	0.51
					Average	13.67	8.26	2.31	9.81	0.60	1.21	0.63	0.55
					Standard Deviation (s)	0.5037	0.4465	0.0573	0.9733	0.0531	0.0927	0.0189	0.0419
					**Coefficient of Variance (CV)**	**3.68**	**5.40**	**2.48**	**9.92**	**8.81**	**7.66**	**2.98**	**7.57**

Table S2. Recovery data.

**Recovery**												
ID	NMR Spectrum	Sampling Site	Provenance	Clone	Abietic acid	Neoabietic acid	Dehydroabietic acid	Palustric acid	Levopimaric acid	Pimaric acid	Sandaracopimaric acid	Isopimaric acid
799-21B	Ioa339	3	Feneos	21	91.29	95.92	88.71	93.40	94.44	93.80	90.91	86.76
578-36A	Ioa340	2	Parnonas	36	88.99	92.50	89.75	88.19	97.64	90.24	88.46	86.18
821-47B	Ioa341	3	Taigetos	47	90.49	92.13	87.90	89.91	80.11	94.21	96.84	89.16
				Average	90.26	93.52	88.78	90.50	90.73	92.75	92.07	87.37
				Standard Deviation (s)	0.9535	1.7043	0.7573	2.1656	7.6221	1.7867	3.5186	1.2892
				**Coefficient of Variance (CV)**	**1.06**	**1.82**	**0.85**	**2.39**	**8.40**	**1.93**	**3.82**	**1.48**
